# Enrichment and Purification of Polyphenol Extract from *Sphallerocarpus gracilis* Stems and Leaves and *in Vitro* Evaluation of DNA Damage-Protective Activity and Inhibitory Effects of α-Amylase and α-Glucosidase

**DOI:** 10.3390/molecules201219780

**Published:** 2015-12-02

**Authors:** Tingting Ma, Xiangyu Sun, Chengrui Tian, Jiyang Luo, Cuiping Zheng, Jicheng Zhan

**Affiliations:** 1 College of Food Engineering and Nutritional Science, Shaanxi Normal University, Xi’an 710062, China; mtt_smile@163.com (T.M.); 18191271130@163.com (C.Z.); 2 College of Food Science and Nutritional Engineering, China Agricultural University, Beijing 100083, China; sunyu7459@cau.edu.cn; 3 National HACCP Center, Chinese Academy of Inspection and Quarantine, Beijing 100123, China; luojy@snnu.edu.cn

**Keywords:** SGslP, purification, phenolic composition, oxidative DNA damage, enzyme inhibition

## Abstract

An efficient preparative separation method for *Sphallerocarpus gracilis* stems and leaves polyphenols (SGslP) was established in this study. An X-5 macroporous adsorption resin was selected for the purification of the SGslP, and the polyphenol content of the purified SGslP (PSGslP) was increased 5.11-fold from 8.29% to 42.38% after one treatment run. The chemical composition of the PSGslP was analyzed by HPLC-MS/MS, and the predominant compounds were found to be luteolin-7-glucoside, acacetin-7-acetyglycoside and its isomers. In addition, the PSGslP was evaluated *in vitro* to determine the DNA damage-protective activity and inhibitory effects of α-amylase and α-glucosidase. The results indicated that the PSGslP exhibited significant protective activities against both ROO• and •OH radical-induced DNA damage. Moreover, the PSGslP exerted a dose-dependent inhibition effect on α-glucosidase but no inhibitory effect on α-amylase. These findings indicate that the *Sphallerocarpus gracilis* stems and leaves are good natural sources of antioxidants and are potent inhibitors of α-glucosidase activity and are potential anti-diabetic inhibitor.

## 1. Introduction

*Sphallerocarpus gracilis* (*S. gracilis*) is a perennial member of the *Apiaceae* family that is widely distributed in China, Mongolia, the eastern region of Siberia and the Far East area of the former Soviet Union. In China, it is an edible and medicinal plant that grows mainly in cold, high-altitude areas, such as Inner Mongolia and Heilongjiang, Qinghai, Gansu, and Tibet Provinces of China [1,2]. According to ancient records, the entire *S. gracilis* plant was generally used to treat rheumatic arthritis, tetanus and ulcers [3] and has several health-promoting effects, such as clearing and activating the channels and collaterals, soothing the liver, strengthening the spleen, nourishing the blood and improving the level of vital energy when used in traditional Chinese medicine [4].

Recent studies on *S. gracilis* have attracted increasing attention due to its potential biological functions. As demonstrated by a review of the literature, these previous studies on *S. gracilis* mainly focused on the extract technology of bioactive compounds [5], chemical composition [2,3], pharmacological activities and biological activities of the effective components in *S. gracilis* roots and seeds [1,2,3,4]. As in ancient records, the entire *S. gracilis* plant was a functional food that can be used as a medicinal herb for health promotion and disease therapy. In many places in China, such as the Gansu province, it is a traditional health food in the countryside, including its root, stems, leaves, and seeds. In recent years, the root and seed of *S. gracilis* are being used by the modern food industry (e.g., as a functional drink and candy) [1]. However, to the best of our knowledge, there are few available reports concerning the stems and leaves of *S. gracilis*. Except as a traditional family food, most of these materials are discarded as agricultural waste or used as firewood by farmers. In our previous test, we found that the phenol content in *S. gracilis* stems and leaves was higher. Because plant polyphenols have a variety of significant biological activities, such as antitumor [6], antioxidant [7], anti-atherosclerosis [8,9], anti-radiation [10,11], antimicrobial activity [12], and anti-hyperglycemic effects [13] and the prevention of cardiovascular disease and other pathologies, we aimed to extract and separate the phenolics from *S. gracilis* stems and leaves and further evaluate their potential biological activities to make the most of these deserted agricultural resources.

The methods used for the extraction of plant polyphenols mainly include organic solvent extraction, ultrasound-assisted extraction, microwave-assisted extraction and enzymatic extraction [14,15]. After extraction, processes of separation and purification are necessary if the phenolics need to be further purified. The literature discusses several methods for the separation of plant polyphenols, such as liquid–liquid extraction, chromatography, ion exchange, membrane filtration and resin adsorption [16,17]. Comparatively, macroporous resins are a type of less-expensive chromatographic materials for the separation of small-molecule natural products based on their molecular weights and polarities. These resins feature a high loading capacity and can be reused many times and have recently been widely used for the separation and purification of bioactive constituents from functional foods [16,17].

Therefore, in the present study, a simple and efficient process was developed for the preliminary separation and purification of the *Sphallerocarpus gracilis* stems and leaves polyphenols (SGslP) with the optimal resin. First, eight macroporous resins with different chemical and physical properties were used to select the optimal resin based on comparisons of their adsorption capacities and desorption ratios for SGslP. Then, various parameters influencing the adsorption and desorption properties of SGslP were optimized, and the experimental equilibrium data at different temperatures were fitted to Langmuir and Freundlich isotherms. Furthermore, the major constituents of the purified SGslP (PSGslP) were identified and quantified by HPLC-MS/MS to gain insights into the compounds responsible for the potential biological activities of the PSGslP. Additionally, the PSGslP was subjected to *in vitro* evaluations to determine its DNA damage-protective activity and the inhibitory effects of α-amylase and α-glucosidase, and we hope that this study will prove helpful for the further exploitation and utilization of this resource.

## 2. Results and Discussion

### 2.1. Static Adsorption and Desorption

#### 2.1.1. Selection of Optimal Resin

According to the “like dissolve like” rule, phenolic compounds usually contain a phenyl group with non-polar and polar multi-hydroxyl groups, and thus, either non-polar resins or polar resins can be applied to the adsorption of polyphenols. Hence, eight macroporous resins ranging from non-polar to polar were employed for the enrichment of the SGslP. As depicted in Table 1, the adsorption capacity (51.26 mg/g) and desorption ratio (84.80 mg/g) of the X-5 resin were higher than those of the other resins in values, though it is not significantly different to LS-305 and NKA-9. The LS-46D resin showed the lowest adsorption capacity, whereas the NKA-2 showed the lowest desorption ratio in values, though it is not significantly different to D4020 and LS-305. This finding indicated that the existence of differences in the adsorption capacities and desorption ratios between the different resins. Because the X-5 resin presented better adsorption and desorption properties, it was selected for the separation of the SGslP.

**Table 1:** Physical properties and results of adsorption capacities and desorption ratios for different macroporous resins.

Resin	Surface Area (m^2^/g)	Average Pore Diameter (Å)	Polarity	Moisture Content (%)	Adsorption Capacity (mg/g)	Desorption Ratio (%)
X-5	500–600	290–300	Non-polar	60.1	51.26 ± 1.45 ^a^	84.80 ± 2.44 ^a^
D4020	540–580	100–105	Non-polar	75.2	43.23 ± 2.46 ^bc^	78.09 ± 3.44 ^ab^
D101	400–520	200–300	Non-polar	65.8	43.88 ± 2.34 ^b^	80.55 ± 3.47 ^a^
AB-8	480–520	130–140	Polar	65.4	43.99 ± 1.97 ^b^	81.65 ± 3.02 ^a^
LS-46D	400–500	150–250	Polar	59.3	40.67 ± 2.81 ^c^	84.74 ± 2.65 ^a^
LS-305	400–500	150–250	Polar	70.9	49.62 ± 2.72 ^a^	79.71 ± 3.67 ^ab^
NKA-9	250–290	155–165	Polar	61.4	48.65 ± 2.49 ^a^	80.45 ± 4.29 ^a^
NKA-2	160–200	145–155	Polar	72.7	43.05 ± 3.15 ^bc^	71.88 ± 1.35 ^b^

The values are the means ± standard deviations (SD) from triplicate measurements, and different letters (a, b, c) in each of the columns indicate that the values are significantly different (*p* < 0.05).

#### 2.1.2. Static Adsorption and Desorption Kinetics on X-5

**Figure 1:**
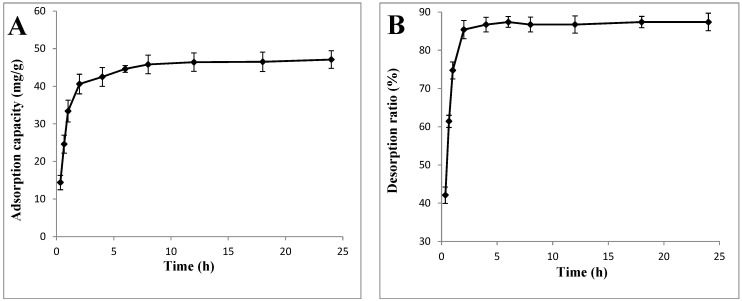

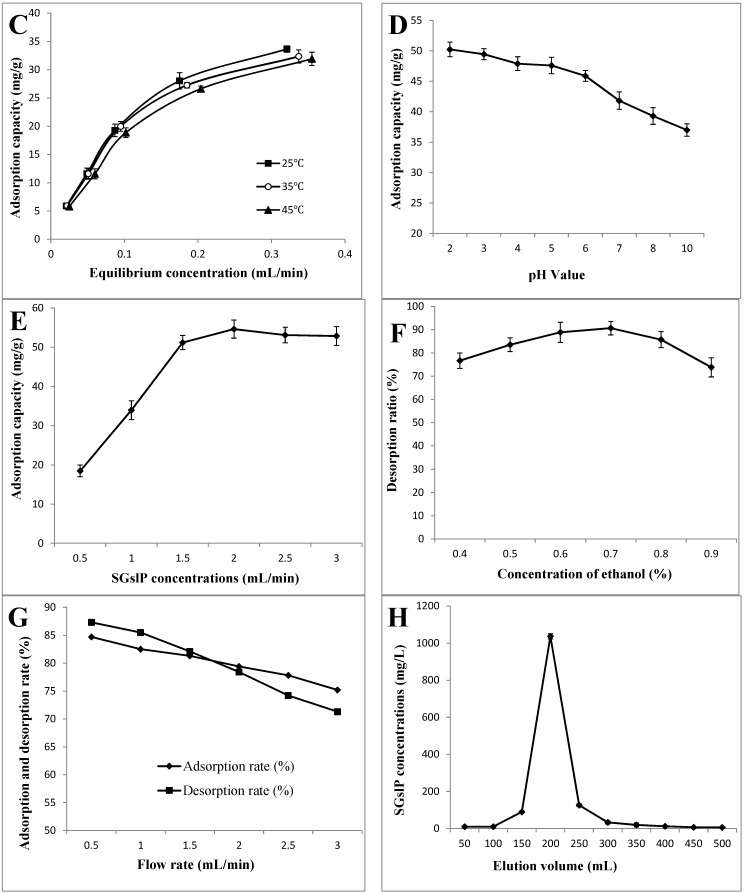
Adsorption and desorption properties of the X-5 resin: (**A**) static adsorption curve; (**B**) static desorption curve; (**C**) adsorption isotherms; (**D**) effect of pH value on the adsorption capacity; (**E**) effect of the concentration of SGslP on the static adsorption; (**F**) effect of the ethanol concentration on the desorption ratio; (**G**) effect of the flow rate on the adsorption capacity/desorption ratio n; and (**H**) dynamic desorption curve of the *Sphallerocarpus gracilis* stems and leaves polyphenols (SGslP).

Static adsorption and desorption experiments were conducted at 25 °C. In the kinetic adsorption test (Figure 1A), the adsorption capacity of the X-5 resin rapidly increased during the first 2 h. After 2 h, the adsorption level did not show any further significant changes, which suggested that the adsorption equilibrium occurred at 2 h, when the adsorption capacity was 40.60 mg/g. In general, the adsorption capacity increased transiently with the adsorption time before reaching the adsorption equilibrium [18], and our results were consistent with those of previous studies [18]. As shown by the desorption kinetics (Figure 1B), the SGslP absorbed into the resin were desorbed effectively by 70% ethanol solution, and the desorption ratio increased rapidly during the initial stage (within 2 h). After 2 h, the desorption ratio did not change significantly, and equilibrium was obtained when the desorption ratio was 85.41%. The kinetic adsorption and desorption properties of the SGslP by the X-5 resin showed a similar tendency to those exhibited by other terrestrial plant polyphenols on macroporous adsorption resins [19]. 

#### 2.1.3. Adsorption Isotherms

Adsorption isotherms can reveal qualitative information regarding the interaction between an adsorbent and an adsorbate as well as the specific effect of the equilibrium concentration of the adsorbate on its degree of accumulation onto the adsorbent surface at a constant temperature. Thus, the process and mechanism of adsorption can be inferred from the adsorption isotherms [15]. The Langmuir and Freundlich equations are the most popular isotherms and are frequently used for the description of adsorption isotherms because of their relative simplicity and reasonable accuracy [20]. The Langmuir isotherm is normally used with an ideal assumption of monolayer adsorption on a homogeneous surface. It is based on two assumptions: (1) the forces of interaction between the absorbed molecules are negligible and (2) no further absorption can take place at a site once it is occupied by a molecule [21]. A Freundlich isotherm is usually suitable for non-ideal adsorption on heterogeneous surfaces. The heterogeneity is caused by the presence of different functional groups on the surface and several interactions. The Freundlich model assumes that the presence of many types of sites acting simultaneously, each with a different free energy of sorption, and that there is a large amount of available sites, which indicates the diversity of free energies associated with the adsorption of the multiple components of a heterogeneous adsorbent [15].

As shown in Figure 1C, the equilibrium adsorption isotherm of the SGslP on the X-5 resin was analyzed at 25, 35 and 45 °C, and the initial volumes of the SGslP solutions were 5, 10, 20, 30 and 40 mL. The adsorption capacity increased with an increase in the equilibrium concentration of SGslP, and the equilibrium adsorption isotherms at 25, 35, and 45 °C showed analogous tendencies. According to the type of equilibrium adsorption isotherm, the isotherm on the X-5 resin confirmed preferential adsorption. All of the model parameters and coefficients of determination (R^2^) are listed in Table 2.

**Table 2:** Adsorption isotherm equations and parameters of SGslP on X-5 resin.

Temperature	Parameter	25 °C	35 °C	45 °C
Langmuir equation	Linear equation	C_e_/Q_e_ = 0.0198C_e_ + 0.0030	C_e_/Q_e_ = 0.0213C_e_ + 0.0031	C_e_/Q_e_ = 0.0216C_e_ + 0.0035
	Q_0_ (mg/g)	50.51	46.95	46.30
	K_ad_ (mL/mg)	6.59	6.87	6.97
	R^2^	0.9912	0.9906	0.9939
Freundlich equation	Linear equation	LnQ_e_ = 0.6529lnC_e_ + 4.3998	LnQ_e_ = 0.6529lnC_e_ + 4.3998	LnQ_e_ = 0.6529lnC_e_ + 4.3998
	K_f_ (mg/g)	81.43	74.79	71.77
	1/n	0.6529	0.6347	0.6555
	R^2^	0.9700	0.9641	0.9707

As observed in Table 2, the related coefficient of the Langmuir equation was higher than that of the Freundlich equation at the same temperature, indicating that the adsorption properties of the SGslP on the X-5 resin were better fitted to the Langmuir model than the Freundlich model. In addition, the higher related coefficient obtained for the Langmuir equation indicted that the adsorption process tended to exhibit monolayer sorption. Generally, in the Freundlich model, the adsorption process is easy to perform when the value of 1/n is less than 1, whereas it is difficult to obtain if the value is greater than 1 [15]. All three values of 1/n presented in Table 2 are less than 1, suggesting that the X-5 resin is appropriate for the enrichment of the SGslP. Additionally, the adsorption capacities decreased as the temperature increased within the temperature range investigated, which indicated that the adsorption process was a thermo-positive process [15]. Similar results were obtained by Wang *et al.* [18]. Therefore, we chose 25 °C for the following experiments.

### 2.2. Effects of Different Parameters on the Adsorption Capacity and the Desorption Ratio of X-5 Resin

#### 2.2.1. pH Values

The pH value of the sample solution is a critical parameter influencing the adsorption properties, which influences the extent of solute ionization, thus affecting the affinity between the solutes and the solutions [18]. The effect of the pH value on the adsorption capacity of the X-5 resin is shown in Figure 1D. In general, the adsorption capacity decreased as the pH value increased, particularly when the pH value was higher than 6. In contrast, no obvious changes on the adsorption capacities were obtained at pH values lower than 5. A similar result was also found by Sun *et al.* [15]. The phenolic molecules were slightly polar and acidic, which make them sensitive to the pH value. Under low pH conditions, the polyphenols exist as molecules that are easily identified and adsorbed by the resin. In contrast, under relatively high pH conditions, the polyphenols may undergo ionization and thus exist as ions, which are more difficult to adsorb [22]. The highest adsorption capacity (50.26 mg/g) was obtained at pH 2.0; therefore, a pH value of 2.0 was chosen as the optimal pH value for the following tests.

#### 2.2.2. Sample Concentrations

As shown in Figure 1E, the adsorption capacity increased with an increase in the sample concentration when the concentration was less than 2.0 mg/mL, whereas the adsorption capacity basically remained stable when the concentration was higher than 2.0 mg/mL. At a low concentration, the adsorption capacity increased as the concentration increased because the number of active sites related to the polyphenols increased. However, with further concentration increases, more impurities were adsorbed on the X-5 resin, resulting in competition for active sites between the polyphenols and the impurities and thereby leading to a slight decrease in the adsorption capacity [16]. The highest adsorption capacity (54.65 mg/g) was observed when the sample concentration was 2.0 mg/mL; therefore, 2.0 mg/mL was selected for the following tests.

#### 2.2.3. Ethanol Concentrations

As shown in Figure 1F, the desorption ratio increased as the concentration increased from 40% (*v*/*v*) to 70% (*v*/*v*), and the highest desorption ratio of 90.67% was observed when the concentration was 70% (*v*/*v*). The desorption ratio decreased gradually as the concentration continued to increase. Similar results were also found by Yin *et al.* [23]. Polyphenols cannot dissolve in low ethanol concentrations, whereas some impurities, such as rutin and phlorizin, are desorbed at high ethanol concentrations [15]. Therefore, an ethanol concentration of 70% was selected as the optimal ethanol concentration.

### 2.3. Dynamic Adsorption and Desorption

#### 2.3.1. Effect of Flow and Elution Speed on the Adsorption Capacity and Desorption Ratio of the X-5 Resin

The liquid flow rate affected the reaction between the solute and the resin and further affected the adsorption capacity and desorption ratio of the resins [16]. As shown in Figure 1G, both the adsorption capacity and the desorption ratio decreased gradually with an increase in the flow rate, Similar results were found by Jia *et al.* [24] and Ma *et al.* [25]. Higher feeding speeds resulted in a portion of the polyphenols leaking out without being adsorbed by the resins. Similarly, at a higher elution speed, parts of the strippant effused directly without sufficiently eluting the resin. Taking the yield and productivity effect into consideration, we selected a feeding speed of 1.5 mL/min and an eluting speed of 1.5 mL/min for the following tests.

#### 2.3.2. Dynamic Desorption Curve on the X-5 Resin

The dynamic desorption curve on the X-5 resin was obtained based on the elution volume and the concentration of eluted polyphenols. As shown in Figure 1H, the adsorbate could be eluted away from the resin by approximately 400 mL of strippant, and the solution eluted at 150–250 mL determined to be the solution containing most of the polyphenols. When the elution volume reached 400 mL, the concentration of polyphenols in the eluted solution was markedly reduced, showing that the majority of the polyphenols had been eluted. Therefore, based on a comprehensive consideration of both effectiveness and economization, the afore-mentioned 400 mL of eluted solution was collected. In addition, as determined by the FC method, the polyphenol content of the SGslP increased 5.11-fold from 8.29% to 42.38% after one treatment run with the X-5 resin.

#### 2.3.3. Qualitative and Quantitative Analysis of the PSGslP

**Figure A1:**
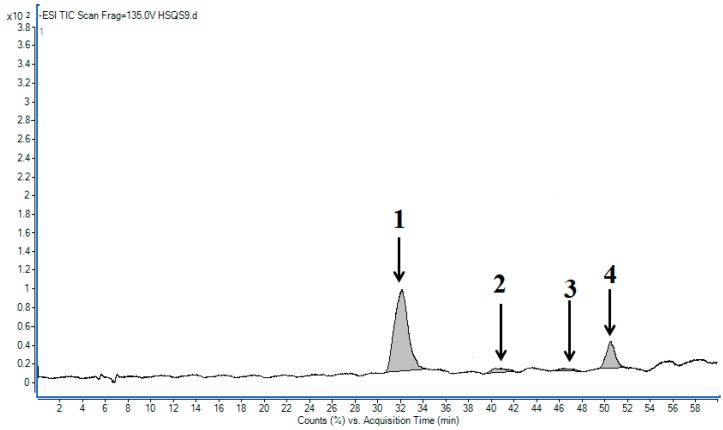
HPLC chromatogram of the PSGslP. The peaks represent (1) luteolin-7-glucoside; (2) acacetin-7-acetyglycoside; (3) acacetin-7-acetyglycoside; and (4) acacetin-7-acetyglycoside.

After the samples were purified under the optimal conditions, HPLC-MS/MS methods were used to separate and identify the phenolic compounds in the PSGslP. As shown in Figure A1 and Table 3 and been reported in our previous report [26], four phenolic compounds were identified by comparing their peak retention times (Rt) and fragmentation profiles with those of standard compounds or published data [4,27,28] in the PSGslP. The compound in peak 1 was identified as L7G, while the compounds in peaks 2, 3 and 4 were tentatively identified as acacetin-7-acetyglycoside, and these compounds are isomers. Due to the lack of a corresponding standard substance, the contents of acacetin-7-acetyglycoside and its isomers were quantified as milligrams of L7G equivalents per gram of dry weight (mg L7G/g DW). Additionally, the major phenolic compounds in the PSGslP were 3.16 mg/g of luteolin-7-glucoside and 0.99 mg L7G/g of acacetin-7-acetyglycoside and its isomers. In contrast, in our previous studies [1,4], the major phenolic compounds of *S. gracilis* roots were found to be caffeic acid, *p*-coumaric acid, ferulic acid and chlorogenic acid, and the major phenolic compounds of *S. gracilis* seeds were found to be chlorogenic acid, L7G and dicaffeoylquinic acid glucoside. These results indicate that the phenolic compound compositions of different parts of the plant are different, which may be due to differences in the biosynthesis and catabolism of phenolics in these structures [4].

**Table 3:** Content and mass spectrum data of the identified phenolic compounds in the purified SGslP (PSGslP).

Parameter	Peak			
	1	2	3	4
Rt (min)	30.66	39.44	41.79	48.87
MW	448	490	490	490
MS (*m*/*z*)	447 [M − H]^−^	489 [M − H]^−^	489 [M − H]^−^	489 [M − H]^−^
MS^2^ (*m*/*z*)	285	285	285	285
Identified compound	Luteolin-7-glucoside	Acacetin-7-acetyglycoside	Acacetin-7-acetyglycoside	Acacetin-7-acetyglycoside
Content ^a^ (mg L7G/g DW)	3.16 ± 0.25	0.21 ± 0.02	0.11 ± 0.01	0.67 ± 0.03

^a^ The values were quantified as mg of luteolin-7-glucoside equivalents/g DW and are expressed as mean values ± SD (*n* = 3).

#### 2.3.4. DNA Damage-Protective Activity of the PSGslP

PBR322 plasmid DNA is a type of circular negatively supercoiled DNA. The open circular DNA will form after the cleavage of one phosphodiester linkage, whereas linear DNA will form when the cleavage of two phosphodiester linkages; therefore, open circular DNA is a sign of a single-strand break, whereas linear DNA indicates a double-strand break. These three forms of DNA molecules exhibit different flowing speeds in an electric field, and supercoiled DNA molecule is the fastest, followed by linear DNA and then open circular DNA. The harmful free radicals produced in the system could result in plasmid DNA damage to different extents, a higher proportion of the supercoiled form indicates less damage to the plasmid DNA. Therefore, based on the proportion of supercoiled DNA, one can evaluate the protective activity of antioxidants against DNA oxidative damage [4,29].

**Figure 2:**
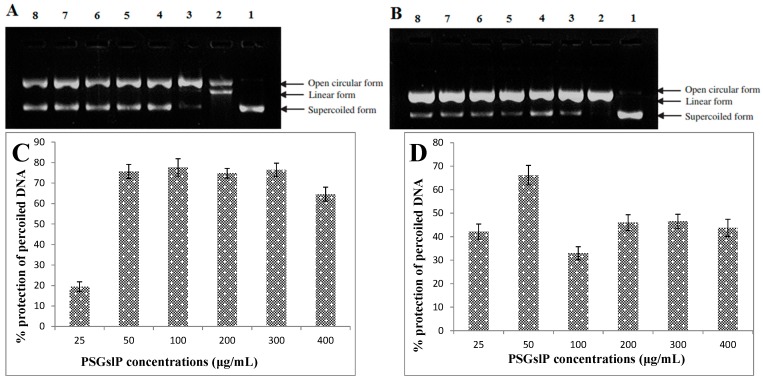
DNA damage-protective activity of the PSGslP. (**A**) Protective activity of the PSGslP against ROO• radical-induced DNA damage. Lane 1, the native DNA; lane 2, DNA treated with AAPH; and lanes 3–7, DNA treated with varying concentrations of the PSGslP (25, 50, 100, 200, 300, and 400 (μg/mL), respectively) in the presence of AAPH; (**B**) Protective activity against •OH radical-induced DNA damage. Lane 1, the native DNA; lane 2, DNA treated with 1 mM FeSO_4_ and 1 mM H_2_O_2_; and lanes 3–8, DNA treated with varying concentrations of the PSGslP (25, 50, 100, 200, 300, and 400 μg/mL, respectively) in the presence of 1 mM FeSO_4_ and 1 mM H_2_O_2_; (**C**) Densitometric quantification of the protective activity against ROO• radical-induced DNA damage; (**D**) Densitometric quantification of the protective activity of the PSGslP against •OH radical-induced DNA damage.

As shown in Figure 2A,B, the normal pBR322 plasmid DNA is mainly composed of the supercoiled form (Figure 2A,B, lane 1), the supercoiled form of DNA is converted into the open circular and linear forms (Figure 2A,B, lane 2) after oxidative damaged by ROO• or •OH radicals, indicating that these harmful free radicals can cause both single-strand and double-strand DNA breaks. However, linear DNA molecules were not observed after the addition of different concentrations of the PSGslP (Figure 2A,B, lane 3–8), indicating that the PSGslP can protect against double-strand DNA breaks mediated by ROO• or •OH radicals.

To further study the DNA damage-protective activity of the PSGslP, we used a gel imaging system, and the test results were performed by the semi-quantitative analysis. The DNA damage protective activity of PSGslP can be characterized by the protection rate (%) such that a higher protection rate indicates a stronger protective activity of the PSGslP. As shown in Figure 2C,D, the PSGslP exerted different degrees of DNA damage-protective activity within the tested concentration range. As a PSGslP concentration of 0.1 mg/mL, the protection rate against ROO• and •OH-induced DNA damage was less than 50%, namely 42.14% and 19.47%, respectively, whereas at a concentration of 0.2 mg/mL, the protection rate was significantly increased to 75.63% and 66.19%, respectively. As the concentration continued to increase, the protection rate did not present an upward trend but remain at a relatively stable level. Additionally, it was noteworthy that the inhibitory activity of the PSGslP against the two radicals is different: the PSGslP exerted more potent inhibitory activity against ROO• radical-induced damage than •OH radical-induced damage on the whole. This difference is mainly due to the fact that •OH and ROO• constitute two different types of free radical systems, and the mechanisms of interaction between the PSGslP and the two types of free radicals were different [4].

#### 2.3.5. Inhibitory Effects of α-Amylase and α-Glucosidase

**Figure 3:**
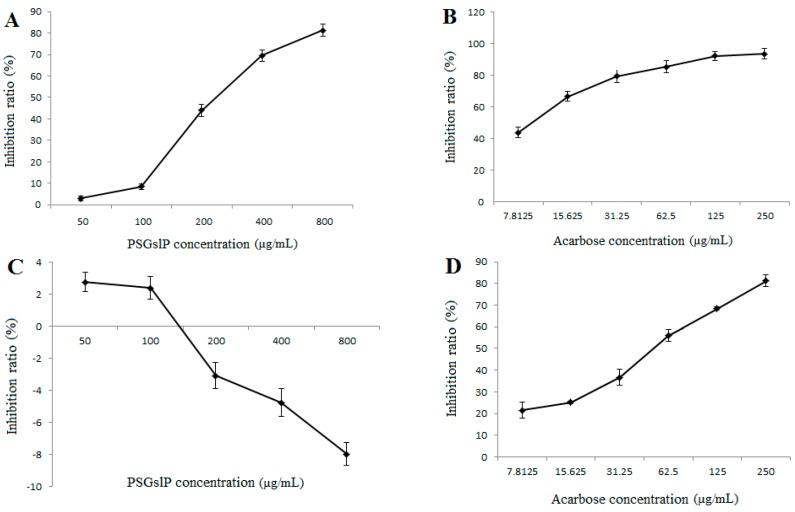
The inhibitory effects of PSGslP on α-amylase and α-glucosidase. (**A**) The inhibitory effect on α-glucosidase; (**B**) The inhibitory effect of acarbose on α-glucosidase; (**C**) The inhibitory effect on α-amylase; (**D**) The inhibitory effect of acarbose on α-amylase.

α-amylase and α-glucosidase inhibitors belong to a class of compounds that aid the control of diabetes by diminishing the absorption of glucose [30]. Recently, the identification of α-amylase and α-glucosidase inhibitors from the natural products has become the focus of researchers. As shown in Figure 3A,B, both the PSGslP and acarbose (positive control) inhibited α-glucosidase activity in a dose-dependent manner over the tested concentration range, and the IC_50_ values of the inhibitory effects of the PSGslP and acarbose on α-glucosidase were 278.68 and 8.88 µg/mL, respectively. As expected, acarbose showed the lowest IC_50_, establishing its excellent performance as an α-glucosidase inhibitor. In general, the PSGslP presented a weak inhibition of α-glucosidase compared with acarbose, but at a concentration of 800 µg/mL, the PSGslP exhibited higher inhibitory activities on α-glucosidase (81.22%). Interestingly, as shown in Figure 3C, it is obvious that the PSGslP barely showed any inhibitory activities on α-amylase and even showed a promoting effect at the higher concentrations tested. However, under the same condition, the positive control acarbose also showed excellent performance as an α-amylase inhibitor (Figure 3D). This difference is mainly due to the fact that α-amylase and α-glucosidase are two different types of amylases, and the mechanisms of interaction between the PSGslP and these two types of amylases are different.

## 3. Experimental Section

### 3.1. Reagents

Luteolin-7-glucoside (L7G), gallic acid, α-amylase from human saliva (E.C.3.2.1.1, 100 U), 4-nitrophenyl-α-d-glucopyranoside (pNPG), α-glucosidase (E.C.3.2.1.20, 100 U) and Folin–Ciocalteu reagent were obtained from Sigma Chemical Co. (St. Louis, MO, USA). Methanol (HPLC-grade) was purchased from Fisher Scientific Co. (Fair Lawn, NJ, USA). All the other chemicals used in the experiments were of analytical grade.

### 3.2. Adsorbents

Macroporous resins, including X-5, D4020, LS-305, LS-46D, D101, NKA-9, NKA-2, and AB-8, were provided by LanShen Special Resin, Ltd. (Xi’an, Shaanxi, China) and Bonchem Co., Ltd. (Cangzhou, Hebei, China). According to the manufacturer’s specifications, the resins were pretreated successively with 1 mol/L HCl and NaOH solutions to remove the monomers and porogenic agents trapped inside the pores during the synthesis process and then dried at 60 °C under vacuum. The dried resins were soaked with 95% ethanol for 12 h and subsequently washed with distilled water thoroughly before the experiments. Their physical properties are listed in Table 1.

### 3.3. Plant Material and Preparation of SGslP Extracts

The *S. gracilis* stems and leaves were collected from Shandan House Husbandry Farm, which have given permission to this study (Gansu, China; latitude, 38°77′N; longitude, 101°08′E; altitude, 2700 m), on July 2013. The SGslP were extracted based on a previously described procedure with minor modifications [4]. 200 g of a powder prepared from defatted *S. gracilis* stems and leaves were extracted three times (each time for 30 min) with 7000 mL of 75% ethanol (*v*/*v*) using an ultrasonic instrument (KQ-600DB, 80 kHz, 200 W, Kunshan Ltd., Jiangsu, China) at 70 °C. The mixtures were then centrifuged at 3000 *g* for 10 min using a centrifuge (LXJ-IIB, Anting Ltd, Shanghai, China), and the supernatants were collected and combined. The solvent was evaporated at 40 °C under reduced pressure in a rotary evaporator (RE 52-99, Yarong Ltd., Shanghai, China) to remove the ethanol. The concentrated aqueous solution was centrifuged at 3000 *g* for 10 min, and 1000 mL of a clear extract supernatant (SGslP solutions) with a total polyphenol concentration of 2.52 mg/mL was obtained.

### 3.4. Determination of the Total Polyphenol Content (TPC)

The TPC was determined using the Folin–Ciocalteu colorimetric method (FC method) with slight modifications [5] and the TPC was calculated as Gallic acid equivalents using a calibration curve with the following equation: Y = 0.1159X + 0.0045 (R^2^ = 0.999).

### 3.5. Static Adsorption and Desorption Test

#### 3.5.1. Resin Selection

All of the macroporous resins were screened through static adsorption tests, which were performed as follows: 2 g of the pretreated resin was placed into a 250-mL triangular flask, and 100 mL of SGslP solution (TPC 1.50 mg/mL) was added. The flasks were continually shaken using a water-bath shaker (SHZ-82B, Zhenjiang Instruments Ltd, Jiangsu, China) at 130 g and 25 °C for 24 h and determined the TPC in the adsorption solution. The resin was then first washed twice with distilled water and then desorbed with 100 mL of 70% ethanol solution at 130 g and 25 °C for 24 h and determined the TPC in the desorption solution. The properties of the eight resins were evaluated based on their adsorption capacities and desorption ratios, and the adsorption capacities and desorption ratios of the individual resins were calculated using the following equations [15]:

(1) $$Q_{e}=\frac{V_{0}\left(C_{0}-C_{e}\right)}{W}$$

(2) $$D=\;\frac{C_{d}V_{d}}{V_{0}\left(C_{0}-C_{e}\right)}$$

where *Q_e_* is the adsorption capacity (mg/g), which represents the mass of adsorbate adsorbed on 1 g of resin at the adsorption equilibrium, *C*_0_ and *C_e_* are the initial and equilibrium concentrations of total polyphenols in solution, respectively (mg/mL), *V*_0_ is the initial volume of solution added into the flask (mL), W is the weight of the resin (g), *D* is desorption ratio (%), *C_d_* is the equilibrium concentration of total polyphenols in the desorption solution (mg/mL), and *V_d_* is the volume of the desorption solution (mL).

#### 3.5.2. Adsorption and Desorption Kinetics

Two grams of the pretreated X-5 macroporous resin was placed into a 250-mL triangular flask, and 100 mL of SGslP solution (1.5 mg/mL) was added. The flask was shaken at 130 g and 25 °C for 24 h. The TPC in the adsorption or desorption process at different time intervals was then determined. The adsorption capacities and desorption ratios were calculated using Equations (1) and (2), respectively.

#### 3.5.3. Adsorption Isotherms

To investigate the effect of the initial concentration and temperature on the total polyphenol absorption, experiments were performed to analyze the adsorption isotherm on the X-5 resin. First, 1.5 mg/mL SGslP solutions in different volumes (5, 10, 20, 30, 40 mL) with 1 g of the pretreated resins were shaken (130 g) at 25, 35 and 45 °C for 12 h. The Langmuir and Freundlich models were then used to evaluate the adsorption behavior between the adsorbate and the adsorbent, and the adsorption isotherms were described by *C_e_* and *Q_e_* as follows [15]:

(3) $$\frac{Q_{e}}{Q_{0}}=\frac{K_{ad}C_{e}}{1+K_{ad}C_{e}}$$

*Q_e_* = *K_f_C_e_*^1/*n*^ (4)

where *Q_e_* is the amount of total polyphenols absorbed per unit mass of X-5 resin (mg/g), *C_e_* is the equilibrium concentration of total polyphenols in solution (mg/mL), *Q*_0_ is the theoretical maximum adsorption capacity (mg/g), *K_ad_* is a constant related to the free energy of adsorption, *K_f_* is the Freundlich constant, which indicates the adsorption capacity, and 1/n is an empirical constant indicating the adsorption intensity of the system.

#### 3.5.4. Effects of Different Parameters on the Adsorption Capacity and Desorption Ratio of the X-5 Resin

The SGslP solutions (1.5 mg/mL) were prepared, and each of them was adjusted to a different pH value (2.0–10.0) using 1.0 mol/L HCl and 1.0 mol/L NaOH solutions. Then, 100 mL of each solution was adsorbed by 2 g of the pretreated resin, and the TPC of the solution was determined after the adsorption had finished. The adsorption capacity was calculated to investigate the relationship between the adsorption capacities and the pH values of the samples.

One hundred milliliters of the SGslP solutions with different concentrations (0.5, 1, 2, 3, and 4 mg/mL) were adsorbed by 2 g of the pretreated resin, and the TPC of the solution was determined after the adsorption process had finished. The adsorption capacity was calculated to investigate the relationship between the adsorption capacities and the sample concentrations.

One hundred milliliters of the SGslP solutions (1.5 mg/mL) was adsorbed by 2 g of resin, and the TPC of the solution was determined after the adsorption had finished. The resins were then washed twice with 100 mL of distilled water to remove the impurities, and 2 g of each of the resins was desorbed by 100 mL of ethanol solution at different concentrations ((50%, 60%, 70%, 80%, and 90% (*v*/*v*)). The desorption ratios were calculated to investigate the relationship between the desorption ratio and the ethanol concentration.

### 3.6. Dynamic Adsorption and Desorption

#### 3.6.1. Effects of Flow Rate on Dynamic Adsorption and Desorption

Dynamic adsorption and desorption experiments were performed on a glass column (25 mm × 500 mm) which was fully wet-packed with X-5 resin. Five-hundred-milliliter-sample solutions containing a total polyphenol content of 2.0 mg/mL were flowed through the glass column at different flow rates (0.5, 1, 1.5, 2.0, 2.5 and 3 mL/min) to investigate the relationship between the adsorption ratio and the flow rate. The flow rate was controlled by a constant flow pump (HL-2, Huxi Ltd., Shanghai, China). At the adsorption equilibrium, the adsorbed column was eluted with 70% ethanol solution at different flow rates (0.5, 1, 1.5, 2.0, 2.5 and 3 mL/min) to investigate the relationship between the desorption ratio and the flow rate of the strippant.

#### 3.6.2. Dynamic Desorption Curve on the X-5 Resin

Five-hundred-milliliter-SGslP solutions (2.0 mg/mL) were flowed through the glass column at a flow rate of 1.5 mL/min, and after adsorption equilibrium was reached, the adsorbed column was washed with 500 mL of distilled water to remove impurities and then eluted with 70% ethanol solution at a flow rate of 1.5 mL/min to investigate the elution curve.

### 3.7. HPLC-MS/MS Analysis of the PSGslPs

The analysis of the phenolic composition of the PSGslP was as our previous report [18,26], using an Agilent 1260 HPLC chromatograph system (Santa Clara, CA, USA) equipped with an Agilent 6460 triple quadrupole mass spectrometer and interfaced with an Agilent 1100 LC/MSD Ion Trap mass spectrometer (MS) equipped with an electrospray interface (ESI). Separation was performed using a reversed-phase C18 column (Merck LiChroCART® 250–4, 5 μm, 250 mm × 4.6 mm, Darmstadt, Germany) protected by a guard column composed of the same material. The injection volume was 10 μL, while using the mobile phase consisted of solvent A (methanol containing 0.3% glacial acetic acid) and solvent B (glacial acetic acid/methanol/H_2_O, 0.3/10/89.7, *v*/*v*/*v*) according to the following gradient elution program for separation: 0–10 min, 30% (A) and then 10–60 min, 30%–44% (A). The detecting wavelength was set to 280 nm. The duration of a single run was 60 min. The mobile phase flow was 0.4 mL/min. The MS instrument was operated in the scanning mode, scanning from *m*/*z* 50 to 1000. The mass spectra were acquired in the negative mode using a source voltage of 4.0 kV, a capillary temperature of 350 °C, and a capillary voltage of −85.5 V. Nitrogen was used as the drying gas and was supplied at 8 L/min and 350 °C.

### 3.8. Evaluation of the DNA Damage-Protective Activities of the PSGslP

#### 3.8.1. Determination of Peroxyl Radical-Induced DNA Damage-Protective Activity

A peroxyl radical-induced plasmid DNA relaxation assay was performed according to Gao *et al.* [4]. Peroxyl radicals were generated by the thermal decomposition of 2-amidinopropane hydrochloride (AAPH). The sample reaction mixture (20 μL) contained 11 μL of 10 mM PBS, 1 μL of plasmid DNA, 3 μL of 50 mM AAPH in PBS, and 5 μL of the tested sample at different concentrations (PBS was substituted for the sample and AAPH in the normal group, and PBS was substituted for the sample in the injured group). The reaction mixture was incubated in the dark at 37 °C for 45 min. AAPH was added immediately before incubation. The reaction was terminated by the addition of 2 μL of loading buffer (0.25% bromophenol blue and 30% glycerol) and electrophoresed on a 1% agarose gel containing 0.5 μg/mL ethidium bromide in Tris/acetate/EDTA gel buffer for 1 h at 70 V. The DNA in the gel was visualized and photographed under ultraviolet light.

#### 3.8.2. Determination of Hydroxyl Radical-Induced DNA Damage-Protective Activity

The ability of the PSGslP to protect supercoiled pBR322 plasmid DNA against hydroxyl radical was estimated using the DNA nicking assay [4,19,29] with minor modifications. Twenty-microliter-sample reaction mixtures contained 10 μL of 10 mM PBS, 1 μL of plasmid DNA, 5 μL of the samples, 2 μL of 1 mM FeSO_4_ and 2 μL of 1 mM H_2_O_2_ (PBS was substituted for the sample, FeSO_4_ and H_2_O_2_ in the normal group, and PBS was substituted for the sample in the injured group) and were then incubated at 37 °C for 30 min. After incubation, 2 μL of a loading buffer (50% glycerol (*v*/*v*), 40 mM EDTA and 0.05% bromophenol blue) was added to stop the reaction, and the reaction mixtures were electrophoresed on a 1% agarose gel containing 0.5 μg/mL ethidium bromide in Tris/acetate/EDTA gel buffer for 50 min (60 V). The DNA in the gel was then visualized and photographed under ultraviolet light.

### 3.9. Evaluation of the Inhibitory Effects of α-Amylase and α-Glucosidase in Vitro

#### 3.9.1. α-Amylase Inhibition Assay

The methods used for the enzymatic inhibition assays performed in this study were adapted from those developed by Kim *et al.* [20,31] with some modifications. Fifty-microliters of PSGslP solutions or acarbose solutions with different concentrations were added to 100 µL 5 U/mL α-amylase solution (in 0.2 M sodium phosphate buffer pH 6.6), and the mixture was blended in a timely manner. Then, 100 µL 1% soluble starch solution (dissolved in sodium phosphate buffer and boiled for 15 min) was added to each tube, and the mixture was incubated at 37 °C for 10 min. The reaction was then terminated by the addition of 0.5 mL of DNS reagent (1% 3,5-dinitrosalicylic acid and 12% sodium potassium tartrate in 0.4 M NaOH). The mixture was then incubated in a boiling water bath for 10 min, and before cooling to room temperature, the mixture was diluted with 3.75 mL of distilled water in an ice bath. The absorbance at 540 nm was measured with a microplate reader. The control contained 100 µL of the buffer solution in place of the a-amylase solution. The background had 100 µL of the buffer solution instead of the soluble starch solution. The inhibitory activity (I) was calculated using the following equation:

(5) $$\text{I}(\%)=(1-\frac{A_{sample}\;-\;A_{background}}{A_{control}})\times 100$$

#### 3.9.2. α-Glucosidase Inhibition Assay

The α-glucosidase inhibitory activity was measured as described by Kim *et al.* [20,31] with slight modifications. Briefly, 0.1 mL of PSGslP solutions or acarbose solutions with different concentrations were added to 0.1 mL of 4 U/mL α-glucosidase solution (in 0.2 M sodium phosphate buffer pH 6.8), and the mixture was blended in a timely manner. Then, 0.1 mL 6 mmol/L pNPG solution (in 0.2 M sodium phosphate buffer pH 6.8) was added to each tube, and the mixture was incubated at 37 °C for 30 min. The reaction was then terminated by the addition of 3 mL 1 mol/L Na_2_CO_3_. The absorbance at 400 nm was then measured with a microplate reader. The control contained 0.1 mL buffer solution in place of the α-glucosidase solution, and the background had 0.1 mL buffer solution instead of the pNPG solution. The inhibitory activity (I) was calculated using Equation (5).

### 3.10. Statistical Analysis

All experiments were performed in triplicate, and the experimental data are expressed as the mean values ± standard deviation (SD). A one-way analysis of variance (ANOVA) and Duncan’s multiple range tests were conducted using DPS software (Ruifeng Ltd., Hangzhou, China, version 7.05) to determine the significance of the differences between groups.

## 4. Conclusions

In conclusion, this study developed a simple and efficient method for the preliminary separation and purification of the SGslP with the X-5 resin. Results indicated that the optimal adsorption parameters were initial concentrations in sample solution of 2.0 mg/mL, pH of 2.0, temperature of 25 °C and feeding speed of 1.5 mL/min; the optimal desorption parameters were ethanol concentration of 70% (*v*/*v*) and eluting speed of 1.5 mL/min. After one treatment run with the X-5 resin, the content of the SGslP increased 5.11-fold from 8.29% to 42.38%. In addition, the adsorption isotherms indicating that the adsorption properties of the SGslP on the X-5 resin were better fitted to the Langmuir model and the adsorption process tended to exhibit monolayer sorption. The PSGslP mainly consisted of L7G, acacetin-7-acetyglycoside and its isomers. Moreover, the *in vitro* activity test of the PSGslP revealed its significant protective activities against both ROO• and •OH radical-induced DNA damage. PSGslP also exerted a dose-dependent inhibition effect on α-glucosidase but no inhibitory effect on α-amylase. These findings indicate that the *S. gracilis* stems and leaves are good natural sources of antioxidants and are potent inhibitors of α-glucosidase activity, which should be further exploited through application of this resource.
